# Spatial and temporal control of expression with light-gated LOV-LexA

**DOI:** 10.1093/g3journal/jkac178

**Published:** 2022-07-25

**Authors:** Inês M A Ribeiro, Wolfgang Eßbauer, Romina Kutlesa, Alexander Borst

**Affiliations:** Department of Circuits-Computations-Models, Max Planck Institute of Neurobiology, 82152 Martinsried, Germany; Department of Circuits-Computations-Models, Max Planck Institute of Neurobiology, 82152 Martinsried, Germany; Department of Circuits-Computations-Models, Max Planck Institute of Neurobiology, 82152 Martinsried, Germany; Department of Circuits-Computations-Models, Max Planck Institute of Neurobiology, 82152 Martinsried, Germany

**Keywords:** light-gated expression, photosensitive, binary expression system

## Abstract

The ability to drive expression of exogenous genes in different tissues and cell types, under the control of specific enhancers, has been crucial for discovery in biology. While many enhancers drive expression broadly, several genetic tools were developed to obtain access to isolated cell types. Studies of spatially organized neuropiles in the central nervous system of fruit flies have raised the need for a system that targets subsets of cells within a single neuronal type, a feat currently dependent on stochastic flip-out methods. To access the same cells within a given expression pattern consistently across fruit flies, we developed the light-gated expression system LOV-LexA. We combined the bacterial LexA transcription factor with the plant-derived light, oxygen, or voltage photosensitive domain and a fluorescent protein. Exposure to blue light uncages a nuclear localizing signal in the C-terminal of the light, oxygen, or voltage domain and leads to the translocation of LOV-LexA to the nucleus, with the subsequent initiation of transcription. LOV-LexA enables spatial and temporal control of expression of transgenes under LexAop sequences in larval fat body and pupal and adult neurons with blue light. The LOV-LexA tool is ready to use with GAL4 and Split-GAL4 drivers in its current form and constitutes another layer of intersectional genetics that provides light-controlled genetic access to specific cells across flies.

## Introduction

Patterned expression of genes is essential for differentiation of distinct cell types during development. Enhancers defining expression patterns have long been used in binary expression systems to study development and function of specific cell types. Binary expression systems couple enhancer-led expression of an exogenous transcription factor to expression of a transgene that sits downstream of promoter sequences exclusively bound by the exogenous transcription factor (del Valle Rodriguez *et al.* 2011). The GAL4-UAS system uses the yeast transcription factor GAL4 under the control of an enhancer that binds upstream activating sequences (UAS), which in turn drive expression of transgenes sitting downstream UAS (Brand and Perrimon 1993). Random insertions of P-elements carrying GAL4 into the genome were used to trap enhancers, with the expression of GAL4 dependent on neighboring regions in the genome (Rubin and Spradling 1982; Spradling and Rubin 1982; Bellen *et al.* 1989; Grossniklaus *et al.* 1989; Wilson *et al.* 1989; Perrimon *et al.* 1991; Lukacsovich, 2001; Venken and Bellen 2012, 2014). More recently, stretches of noncoding genomic DNA carved out of known gene enhancers, or from regions predicted to contain enhancers, have been extensively used to generate large collections of driver lines (Pfeiffer *et al.* 2008, 2010; Jenett *et al.* 2012; Kvon *et al.* 2014; Yáñez-Cuna *et al.* 2014; Tirian and Dickson 2017). Other binary expression systems were added to the fruit fly genetic toolbox. The LexA-LexAop (Lai and Lee 2006) and the QF-QUAS (Potter *et al.* 2010; Riabinina *et al.* 2015) systems rely on exogenous transcription factors and DNA binding sequences, and can be combined with GAL4-UAS, allowing for independent access to multiple cell types in the same organism (e.g. Feng *et al.* 2014, 2020; Sen *et al.* 2017; Ribeiro *et al.* 2018). The spatial resolution, or cell type specificity of binary expression systems, is determined by the enhancer driving expression of the exogenous transcription factor. Given that it is still not possible to design enhancers specific for many cell types (Serebreni and Stark 2021), it is necessary to screen to obtain enhancers specific for the cell type of interest.

Several methods, under the umbrella of intersectional genetics, were developed to further restrict transgene expression in binary systems. The modular nature of the GAL4 activation and DNA-binding domains enables separation of GAL4 into two parts, with each split-GAL4 half placed under the control of a different enhancer (Luan *et al.* 2006). The final transgene expression occurs only in cells that express both split-GAL4 halves, which dimerize through added leucine zipper domains to form a fully functional transcription factor. Existing collections of split-GAL4 lines targeting single neuronal types were established by screening for enhancer pairs that together provide exclusive access to specific cell types of interest (e.g. Wu *et al.* 2016; Dionne *et al.* 2018; Namiki *et al.* 2018; Dolan *et al.* 2019; Schretter *et al.* 2020; Wang *et al.* 2020; Sterne *et al.* 2021). Other powerful methods of restricting expression to single or fewer cells include the recombinase-based systems for stochastic labeling (Lis *et al.* 1983; Golic and Lindquist 1989; Xu and Rubin 1993; Lee and Luo 1999; Hadjieconomou *et al.* 2011; Nern *et al.* 2015; Isaacman-Beck *et al.* 2020), temperature sensitive mutations of Gal80, the repressor of GAL4 (Nogi *et al.* 1977; Lee and Luo 2001; McGuire *et al.* 2003), and use of transcription factors modified to drive transcription in the presence of an ingestible drug (McGuire *et al.* 2004). In addition to providing temporal and spatial control, however, these methods either involve increase in temperature that unleashes a stress response in all cells of the organism (Lindquist 1986), or addition of drugs with potential off-target effects, both of which may affect experimental outcomes.

With several recent advances in optics and laser technology, light is now easily modulated at the level of its spectrum, intensity, and even beam shape (Chen *et al.* 2018). The high spatial and temporal precision of light pulse delivery to living organisms has the potential to take the spatial and temporal resolution of transgene expression to new levels. Several photosensitive proteins have been introduced into exogenous expression systems to amass the advantages of light as a precise trigger (Di Ventura and Kuhlman 2016; de Mena *et al.* 2018; di Pietro *et al.* 2021). Phytochromes (Phy) are sensitive to red and far-red light, and bind the phytochrome-interacting factor (PIF) in presence of light (Yamamoto and Deng 1999). The Photo-GAL4 tool capitalizes on the PhyB and PIF light-dependent interaction to reconstitute a complete GAL4 upon exposure to light (de Mena and Rincon-Limas 2020). To function, Photo-GAL4 requires addition of phycocyanobilin (PCB), a chromophore that is absent in animal cells (Yamamoto and Deng 1999), limiting its applicability (de Mena and Rincon-Limas 2020). The cryptochrome split-LexA system similarly uses cryptochrome 2 and its binding partner, the cryptochrome interacting protein, to gate reformation of split-LexA with blue light (Szuts and Bienz 2000; Chan *et al.* 2015). In ShineGal4, the pMagnet and nMagnet photoswitches derived from the blue light photoreceptor VVD endogenous to *Neurospora crassa* replace the leucine zippers in split-GAL4 halves and heterodimerize upon exposure to light (Kawano *et al.* 2015; di Pietro *et al.* 2021). ShineGal4 functions in several epithelia across developmental stages, with its current form limited to a few drivers.

To circumvent these limitations and expand the photosensitive toolbox in *Drosophila*, we developed a light-gated expression system based on the light, oxygen, or voltage (LOV) domain originally found in oat phototropin 1 (*Avena sativa*) (Christie *et al.* 1998, 1999; Crosson and Moffat 2002) and LexA (Horii *et al.* 1981; Walker 1985; Rhee *et al.* 2000; Masuyama *et al.* 2012), under the control of UAS sequences. LOV-LexA gates expression of transgenes with blue light *in vivo*, in several cell types in larval, pupal, and adult fruit flies. LOV-LexA can be directly crossed to split-GAL4 and GAL4 drivers, adding thus another layer of spatiotemporal control to transgene expression in *Drosophila* that is combinable with existent binary expression systems and transferable to other model organisms.

## Materials and methods

### Plasmids and cloning

The LexA chimeras LexA:GAD, LexA:p65, and LexA:VP16 from the plasmids pBPLexA::GADUw, pBPLexA::p65Uw, and pBPLexA::VP16Uw (Addgene # 26230, 26231, 26232; Gerald Rubin Lab) were mutagenized to change the NLS-like sequence from (2433) GTT ACT GTG AAA CGT CTC AAG AAG CAA GGC AAT (VTVKRLKKQGN) to (2433) GTT ACT GTG AAA GGG CTC GAG AAG CAA GGC AAT (VTVKGLEKQGN) (Rhee *et al.* 2000), using the Q5 site directed mutagenesis kit (New England Biolabs, catalog # E0554S). The resulting modified LexA (mLexA) chimeras were combined through DNA assembly (Gibson *et al.* 2009) with the following components: eLOV (Addgene # 92213; Alice Ting Lab) (Wang *et al.* 2017), SV40 nuclear localizing signal (Pfeiffer *et al.* 2010) and tdTomato (Shaner *et al.* 2004) or GFP (from pJFRC7-20XUAS-IVS-mCD8::GFP, Addgene # 26220; Gerald Rubin Lab) (Pfeiffer *et al.* 2008), or FLAG (amino acid sequence: DYKDDDDK) with a kit (Gibson assembly kit from New England Biolabs, catalog # E5510S). The different combinations were cloned into pJFRC7-20XUAS-IVS-mCD8::GFP (Addgene # 26220) and cut with XhoI (NEB catalog # R0146S) and XbaI (NEB catalog # R0145S) to replace mCD8::GFP and produce pJFRC7-20XUAS-LexA-transactivator-eLOV-tag construct combinations (Supplementary Table 1).

### S2R+ cell culture, transfection, stimulation, fixation, and immunostaining

The *Drosophila* cell line S2R+ (Echalier 1997) was obtained from the Drosophila Genomics Resource Center, supported by NIH grant 2P40OD010949. S2R+ cells were cultured at 25°C in Schneider’s medium (Gibco, cat # 21720-024) containing 10% fetal bovine serum (Gibco, cat # A47668-01) and 1% penicillin–streptomycin (Gibco, cat # 15070-063). To test the various LexA-transactivator-eLOV-tag constructs, listed in Supplementary Table 1, for cell survival and ability to drive expression gated by light, S2R+ cells were transfected with pMET-GAL4 as the driver, the UAS-LexA-transactivator-eLOV-tag test construct, and pJFRC19-13XLexAop2-IVS-myr::GFP (Addgene # 26224) or 13XLexAop-IVS-myr::tdTomato (this study) as the LexAop-led reporters of LexA-transactivator-eLOV-tag transcriptional activity. We used the cotransfection of pMET-GAL4 (Velichkova *et al.* 2010), pJFRC7-20XUAS-IVS-mCD8::GFP (Pfeiffer *et al.* 2008), and pJFRC7-20XUAS-IVS-mCherry (this study) as controls to characterize the transfection efficiency of three constructs simultaneously. Three DNA plasmids, 200–250 ng/µl, were combined with FuGene (Promega, cat # E2311) in Schneider’s media with a proportion of 600–750 ng DNA for 4ul FuGene. The DNA plasmid/FuGene mix was allowed to stand for 30 min to 1 h at room temperature, after which it was added to roughly 1 million cells preplated in a 24-well plate. The metallothionein promoter in the pMET-GAL4 driver (Velichkova *et al.* 2010) is activated by the addition of copper sulfate (CuSO_4_, Sigma-Aldrich Nr. 451657), to a final concentration of 0.75 mM. Presentation of light was initiated 1–3 h after the addition of copper sulfate. Light was delivered in pulses of 30 s of blue LED (from the LED light source of the inverted laboratory microscope LEICA DM IL LED) at 1 Hz (Supplementary Table 3). Cells were fixed with 4% paraformaldehyde overnight at 4°C, between 8 and 10 h after addition of copper sulfate, and processed for immunostaining with standard protocols (e.g. Velichkova *et al.* 2010), with antibodies anti-GFP chicken antibody dilution 1:2,000 (Rockland, catalog # 600-901-215S; RRID: AB_1537403), anti-RFP rabbit antibody dilution 1:2,000 (Rockland, catalog # 600-401-379, RRID: AB_11182807), and anti-FLAG rat antibody dilution 1:300 (Novus Biologicals, catalog # NBP1-06712, RRID: AB_1625981). Secondary antibodies were goat anti-chicken Alexa Fluor 488 1:1,000 (Thermo Fischer Scientific, catalog # A-11039; RRID: AB_2534096), goat anti-rabbit Alexa Fluor 568 1:1,000 (Thermo Fischer Scientific, catalog # A-11011; RRID: AB_143157), and goat anti-rat Alexa Fluor 568 1:1,000 (Thermo Fischer Scientific, catalog # A-11077; RRID: AB_2534121).

### Quantification of signal intensity in cell culture

**Fig. 1.**
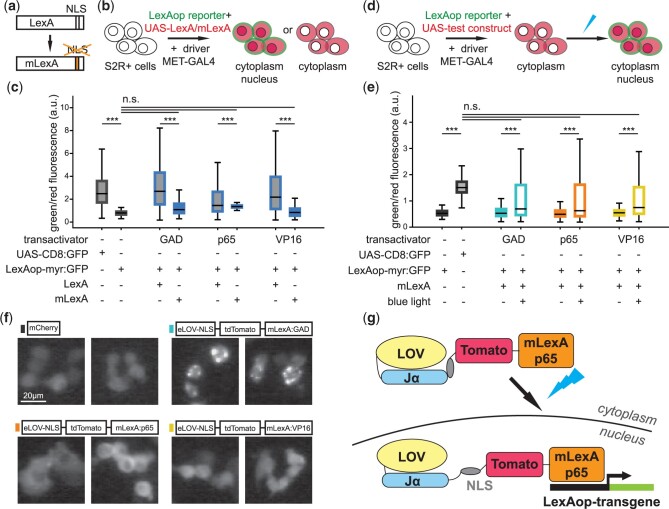
Testing components for a light-gated expression system based on eLOV. a) The NLS-like sequence in *LexA* is mutagenized in *mLexA*. b) S2R+ cell line was used to test whether LexA-transactivator:tdTomato and mLexA-transactivator:tdTomato drive transcription of a LexAop-reporter, *LexAop-myr:GFP*, using the *MET-GAL4* driver. c) The ratio of LexAop reporter *myr:GFP* expression in relation to expression of the different *LexA-* and *mLexA-*transactivator chimeras determined by tdTomato signal. Cotransfection of *UAS-mCherry* and *UAS-CD8:GFP* was used as an approximate measure of coexpression (first bar in the boxplot), whereas cotransfection of *UAS-mCherry* with the LexAop reporter *LexAop-myr:GFP* established the baseline (second bar in the boxplot). The constructs for GAD were *LexA:GAD-tdTomato* and *mLexA:GAD-tdTomato*, for p65 were *LexA:p65-tdTomato* and *mLexA:p65-tdTomato*, and for VP16 were *LexA:VP16-tdTomato* and *mLexA:VP16-tdTomato*. Mutagenizing NLS-like sequence reduces the transcriptional activity of mLexA-transactivator chimeras compared to LexA-transactivator chimeras. At least 200 cells with medium levels of expression of *mCherry* or *tdTomato*, from at least 2 transfections of S2R+ cells are represented for each condition. d) S2R+ cells were transfected with a reporter, *LexAop-myr:GFP*, together with the driver *MET-GAL4* and the test construct, to examine light-gated transcription for test constructs. e) Expression of LexAop reporter *myr:GFP* in relation to expression of mLexA-transactivator chimeras combined with eLOV. Placement of eLOV-nls N-terminal followed by the fluorescent protein tdTomato and the mLexA-transactivator chimera yielded the best signal for cells exposed to pulses of blue light, while maintaining a low reporter signal in cells kept in the dark. As in (b), cotransfection of *UAS-mCherry* with *UAS-CD8:GFP* (second bar in plot) or *LexAop-myr:GFP* (first bar in plot) served as a positive and negative control, respectively. The test constructs were *eLOV-nls-tdTomato-mLexA:GAD* (GAD), *eLOV-nls-tdTomato-mLexA:p65* (p65), and *eLOV-nls-tdTomato-mLexA:VP16* (VP16). At least 200 cells with medium levels of expression of *mCherry* or *tdTomato*, from 2 to 5 transfections of S2R+ cells are represented for each condition. c, e) *** represents *P*-values <0.001; n.s. represents *P*-values >0.05, obtained with Student’s *t*-test. f) Representative examples of S2R+ cells expressing test constructs indicated above the images under the control of *MET-GAL4*. The eLOV-nls-tdTomato-mLexA:GAD forms clusters in the cytoplasm, whereas both eLOV-nls-tdTomato-mLexA:p65 and eLOV-nls-tdTomato-mLexA:VP16 are evenly distributed in the cytoplasm, and sometimes nucleoplasm, like mCherry. g) Schematic representation showing how eLOV-nls-tdTomato-mLexA:p65 (or LOV-LexA) works.

Five images per well, in 24-well plates, were obtained from immuno-stained cells under an inverted fluorescence microscope (Leica DM IL LED), with a 5× objective, with green and red filter cubes. The open-source software CellProfiler (version 4.1.3) (Carpenter *et al.* 2006; McQuin *et al.* 2018) was used to segment individual cells in each image, based on the test construct fluorescent tag signal with the Otsu method, and measure the amount of LexAop reporter in each segmented cell, as a proxy for transcription levels of LexAop reporters by test constructs, LexA-transactivator-eLOV-tag. We used the mean intensity of segmented cells in Cell Profiler, Object MeanIntensity, as a measure of mean pixel intensity per segmented cell, in the green and red channels. This measure is a normalized value by default in Cell Profiler and is plotted in Fig. 1, c and d and Supplementary Fig. 1, b and d. Scripts written in Python (version 3.8, http://www.python.org) were then used to read data values per Object, average all segmented cells across at least 2 independent experiments and plot the data.

### *Drosophila* culture and genetics

Fly stocks obtained from the Bloomington Drosophila Stock Center (NIH P40OD018537) were used in this study. All the strains of the fruit fly *Drosophila melanogaster* used in this study are listed in Supplementary Table 2. Fruit flies were maintained on standard cornmeal-agar medium supplemented with baker’s yeast and incubated at 18 or 25°C with 60% humidity and 12 h light/dark cycle. Males and females were tested indiscriminately throughout experiments. Larvae of the second and third instar were used for tests on fat body with *Cg-GAL4*; 2–4 days after puparium formation (APF), pupae were used for tests in neurons in the central brain and dorsal abdominal oenocytes; adults ranging from 1 to 6 days old were used for tests in adult neurons.

### Light stimulation in fat body

To test *UAS-LOV-LexA*, *UAS-eLOV-nls-tdTomato-mLexA:GAD*, and *UAS-eLOV-nls-tdTomato-mLexA:VP16* constructs in fat body cells, second-to-third instar larvae from crosses with *Cg-GAL4* (Asha *et al.* 2003) were removed from the food, washed in water, and placed in a well with 40 µl of 15% sucrose in water solution; 1 larva per well in a 96-well plate wrapped in aluminum foil to shield larvae from light. Pulses of blue light were delivered to individual wells, with the 96-well plate mounted on an inverted microscope. Light from a blue LED (LEICA DM IL LED) was delivered at 11.7 mW, at 1 Hz for 30 s (Supplementary Table 3). Half the plate was not exposed to light and larvae in such wells served as controls. The fat bodies were dissected 6–12 h after light delivery, fixed, and immunostained with anti-GFP chicken antibody dilution 1:1,000 (Rockland, catalog # 600-901-215S; RRID: AB_1537403) and anti-RFP rabbit antibody dilution 1:2,000 (Rockland, catalog # 600-401-379, RRID: AB_11182807). Secondary antibodies were goat anti-chicken Alexa Fluor 488 1:1,000 (Thermo Fischer Scientific, catalog # A-11039; RRID: AB_2534096) and goat anti-rabbit Alexa Fluor 568 1:1,000 (Thermo Fischer Scientific, catalog # A-11011; RRID: AB_143157). Stained fat bodies were mounted with Vectashield Antifade Mounting Medium (BIOZOL, Ref H-1000) and imaged under a Leica TCS SP8 confocal microscope.

### Light stimulation in neurons

To stimulate neurons in pupal stages, pupae were recovered from vials by adding water to the vial wall to dissolve the glue-binding pupal cases to the pupation site. Pupae aged between 2 and 3 days APF were then dried on a kimwipe tissue and glued on a double-side sticky tape spread on a cover slip (Fig. 3c) that was attached to a microscope slide with plasticine. Adult flies were glued to a custom-made aluminum or plastic plate with a hole, with a diameter ranging from 300 to 400 µm, large enough to expose part of the head of the adult fly and shield the rest of the fly from light. Melted Eicosane 99% (Aldrich 219274-5G) was added to the thorax and part of the head to immobilize the adult fly and shield part of the head from light (Fig. 4f). Such custom holders were mounted on a microscope slide with plasticine, separating the flies from the slide.

Slides bearing pupae or adult flies were then mounted on an upright confocal microscope (Leica TCS SP8) for preprogrammed serial light delivery with the 458-nm laser at 10% power at 5.75 µW (Supplementary Table 3). Each light pulse was composed to 30–50 scans across a depth of 200–400 µm. Using the xyzt mode of Leica software together with position mapping, it was possible to deliver light serially to many pupae or adult flies prepped together. After light delivery, cover slips with pupae were vertically inserted into new food vials and incubated for 2 days for expression in neurons in the fly brain, before dissection. Adult flies were removed from holders after light delivery, by breaking the brittle Eicosane, placed in fresh food vials, and incubated at 25°C for 1–2 days before dissection.

Adult brains were dissected in cold PBS, fixed in 4% PFA for 20–40 min at room temperature, washed in PBS with 0.5% Triton X-100 2 times, incubated with DAPI (Invitrogen, D1306) at dilution 1:3,000 in PBS with 0.5% Triton X-100 for 10 min, washed again, mounted with Vectashield Antifade Mounting Medium (BIOZOL, Ref H-1000), and imaged on the same day under a Leica TCS SP8 confocal microscope.

### Live imaging in oenocytes and neurons

The pupal case covering the most anterior abdomen in the case of oenocytes (*w; 109(2)-GAL4, UAS-CD8:GFP/+; UAS-LOV-LexA/+*), or the head in case of neurons (*w, LexAop-CsChrimson:Venus;+; UAS-LOV-LexA/fru-GAL4)*, of pupae lined up on a double side sticky tape on a slide (see above, Fig. 4a), was removed under low light conditions, or as low as possible since pupal cuticle is transparent. The slide was mounted on a Leica TCS SP8 confocal microscope, and positions for serial imaging were marked. The pupae were then kept in the dark for 30 min before live imaging was initiated.

Oenocytes were first scanned in the red channel alone, followed by exposure to blue light (see Supplementary Table 3). Afterward, oenocytes were imaged in the red channel every 5 min for at least 1 h and 40 min. Oenocytes were imaged one last time in the red and green channels, obtain CD8:GFP signal to delineate the oenocyte cell body, in addition to LOV-LexA. To image *fru+* neurons in pharate adult pupae (4 days APF), a scan in the green and red channels preceded the exposure to light (see Supplementary Table 3), after which pupal heads were scanned in the red and green channels every hour. Most pupae eclosed after 12–14 h under the confocal microscope.

### Quantification of signal intensity in flies

Mounted fat body and brain tissue were imaged under a Leica TCS SP8 confocal microscope with a 20.0× objective, using the lasers 405, 488, and 568 nm to image DAPI, Venus, and tdTomato respectively. The same laser power, gain, and line averaging were used within each experiment to compare fluorescence levels across light and dark conditions. Z-stacks thus obtained were cropped in XY and Z with ImageJ/Fiji (Schindelin *et al.* 2012), to isolate cell bodies expressing the test construct. Z-projections of these crops were loaded with the Scikit-image image processing package into Python (version 3.8, http://www.python.org), to obtain pixel intensity values in a 2-dimensional matrix for each color channel in RGB. The ratio of the mean pixel intensity in green and red channels, or green and blue channels, was used to compare relative fluorescence levels of reporter gene to test constructs.

## Results

### Design of an expression system gated by light

The LOV2 domain of *A. sativa* phototropin 1, AsLOV2, is photosensitive (Harper *et al.* 2003; Lungu *et al.* 2012; Zayner *et al.* 2012; Diensthuber *et al.* 2014). Exposure to blue light causes the Jɑ helix to unfold, thereby freeing its C-terminus (Harper *et al.* 2003). This property arises from interactions with flavin, the blue light-absorbing chromophore present in animal cells, and can be used to expose a small peptide of up to 10 amino-acid residues long, added to or integrated into the Jɑ C-terminus (Huala *et al.* 1997; Christie *et al.* 1998, 1999; Salomon *et al.* 2000; Harper *et al.* 2003). This photosensitive system has been used to cage several peptides in genetic tools, including the nuclear-localizing signal (NLS) to shuttle proteins to the nucleus, the tobacco etch virus protease (TEVp) cleavage site for an integrator of neuronal activity and reporters of protein-protein interactions (Wang *et al.* 2010; Strickland *et al.* 2012; Motta-Mena *et al.* 2014; Niopek *et al.* 2014, 2016; Guntas *et al.* 2015; Yumerefendi *et al.* 2015, 2016; Jayaraman *et al.* 2016; Reade *et al.* 2017; Smart *et al.* 2017; Salinas *et al.* 2018; van Haren *et al.* 2018; Zhao *et al.* 2018; Cavanaugh *et al.* 2020). Recent work employed directed evolution on the native AsLOV2 to develop the evolved LOV (eLOV) that presents improved stability in the dark state due to 3 single-nucleotide mutations (Kim, Wang, *et al.* 2017; Wang *et al.* 2017; Kim *et al.* 2019). We added the short NLS from SV40 (Pfeiffer *et al.* 2010), to make eLOV-nls and regulate availability of the NLS to the cell milieu with blue light (Niopek *et al.* 2014).

To build a transcription factor gated by light, we selected the binary expression system LexA/LexAop (Szuts and Bienz 2000; Loewer *et al.* 2004; Lai and Lee 2006), that is complementary to the widespread GAL4-UAS system and has been successfully incorporated in diverse model organisms (Lai and Lee 2006; Emelyanov and Parinov 2008; Nonet 2020). LexA is a repressor of transcription endogenous to *Escherichia coli* (Horii *et al.* 1981), where it regulates the SOS response (Walker 1985). Addition of an activation domain to the C-terminal of LexA renders such LexA-transactivator chimeras capable of activating transcription of transgenes sitting downstream of the LexA operator (LexAop) (Rhee *et al.* 2000; Lai and Lee 2006). In *Drosophila*, the most common LexA-transactivator chimeras contain the activation domains GAL4 activation domain (GAD, LexA:GAD), p65 (LexA:p65), or VP16 (LexA:VP16) (Rhee *et al.* 2000; Szuts and Bienz 2000; Lai and Lee 2006; Emelyanov and Parinov 2008; Yagi *et al.* 2010). Despite its bacterial origin, LexA carries an NLS-like sequence that allows it to shuttle to the nucleus when expressed in eukaryotic cells (Rhee *et al.* 2000; Pfeiffer *et al.* 2010; Masuyama *et al.* 2012). To make the translocation of LexA to the nucleus solely dependent on eLOV-nls, we mutagenized the NLS-like sequence in the LexA codon optimized for *D. melanogaster* (Pfeiffer *et al.* 2010; Rhee *et al.* 2000) and created a mLexA (Fig. 1a, see *Materials and Methods*). We examined the propensity to translocate to the nucleus of mLexA-transactivator chimeras by transfecting such constructs into the S2R+ *Drosophila* cell line (Echalier 1997), together with the *metallotheionein-GAL4* (*MET-GAL4*) that drives ubiquitous expression upon addition of CuSO_4_ (Velichkova *et al.* 2010), and the reporter *myr:GFP* under control of LexAop sequences (Pfeiffer *et al.* 2010) (Fig. 1b). All three chimeras of unmodified LexA-transactivator drove expression of the *LexAop-myr:GFP* to levels similar to *UAS-CD8:GFP* (Fig. 1c), confirming their ability to shuttle to the nucleus (Rhee *et al.* 2000; Pfeiffer *et al.* 2010; Masuyama *et al.* 2012). In contrast, mLexA-transactivator chimeras led to reduced expression of the reporter transgene (Fig. 1c), confirming that the NLS-like sequence in LexA plays a major role in shuttling LexA to the nucleus.

We combined eLOV-nls with the 3 mLexA-transactivator chimeras, and a fluorescent protein (Shaner *et al.* 2004) placed each combination under the control of UAS and tested their performance in S2R+ cells cotransfected with *MET-GAL4* and *LexAop-myr:GFP* for constructs tagged with a red fluorescent protein or *LexAop-myr:tdTomato* for constructs tagged with GFP (Supplementary Fig. 1, a and c). Several mLexA constructs carrying eLOV-nls at the C-terminal led to the expression of *myr:GFP* in the dark (Fig. 1e and Supplementary Fig. 1, b and d), indicating that NLS is frequently uncaged with eLOV-nls at the C-terminal end, even in the absence of light. On the other hand, many of the mLexA constructs with eLOV-nls N-terminal were unable to drive the expression of *myr:GFP* upon presentation of blue light (Supplementary Fig. 1, b and d). The combinations made with LexA:GAD chimera formed clusters in the cytoplasm irrespective of the fluorescent protein used as a tag (Supplementary Fig. 1, e and h), while most other combinations were homogenously distributed in the cytoplasm and occasionally in the nucleoplasm (Fig. 1f and Supplementary Fig. 1f–j). Of note, cells with high levels of expression of many of the mLexA constructs tested, presented the expression of *myr:GFP* irrespective of the light regime delivered (data not shown), indicating that eLOV is unstable if expressed at high levels, as previously observed (Kim, Wang, *et al.* 2017, 2019). Cells expressing *eLOV-nls-tdTomato-mLexA:p65* and *eLOV-nls-tdTomato-mLexA:VP16* at moderate levels presented no to very little *LexAop-myr:GFP* reporter expression in the dark and displayed an increase in the expression of *LexAop-myr:GFP* upon exposure to blue light (Fig. 1e). These two constructs thus gathered the characteristics necessary for a light-gated expression system and were used to create transgenic flies. Despite its shortcomings, the *eLOV-nls-tdTomato-mLexA:GAD* was also injected since mLexA:GAD is suppressible by Gal80, potentially providing another level of regulation of a light-gated expression system.

### Characterization of eLOV-nls-tag-mLexA chimera constructs in vivo

**Fig. 2.**
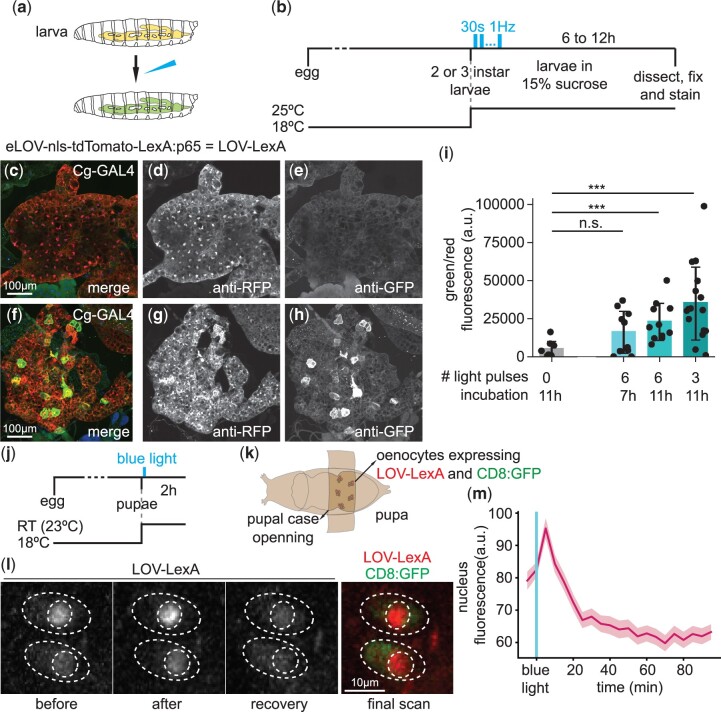
LOV-LexA is gated by light *in vivo*. a) *Drosophila* larvae expressing *LOV-LexA* in the fat body were exposed to blue light and examined for expression of the LexAop reporter, as well as the construct selected for *LOV-LexA*. b) Schematics showing the timeline of the experiment, light regime. Second or young third instar larvae were selected from vials kept at 18°C and transferred to 15% sucrose solution. The first light pulse was delivered immediately after this transfer, with a blue LED on an inverted microscope (Supplementary Table 3). Larvae were placed in the dark at 25°C between light pulses (see text for details), until dissection. Fat bodies expressing *LOV-LexA* with *Cg-GAL4* for second to third instar larvae kept in the dark (c–e) or exposed to 3 30-s pulses of blue LED light at 1 Hz (f–h). Exposure to blue light appears to alter LOV-LexA cellular distribution (d, g) and leads to the expression of *LexAop-CsChrimson:Venus* in fat body cells as detected with anti-GFP antibody (e, h). i) Ratio of fluorescence, measured as pixel intensity in confocal-acquired images, of anti-GFP signal/anti-RFP signal for stained fat bodies from larvae with the genotype *w, LexAop-CsChrimson:Venus; Cg-GAL4/+; UAS-LOV-LexA/+* that were kept in the dark (*N* = 7, representative example in (c)–(e), exposed to 6 light pulses and dissected after 7 h (*N* = 10), or 11 h (*N* = 11), or exposed to 3 light pulses and dissected 11 h later (*N* = 16, representative example in (f)–(h). Varying number of light pulses and the incubation period at 25°C before dissection led us to conclude that LOV-LexA gates expression with light in fat body and that LOV-LexA light-gated expression is highest with 3 light pulses and an 11-h incubation period at 25°C. *** represents *P*-values <0.001, n.s. represents *P*-values >0.05, 2-tailed Mann–Whitney tests. Exposure to blue light leads to an increase in the amount of Venus relative to LOV-LexA levels. j) Schematics showing the timeline of the experiment, light exposure, and functional imaging. k) *Drosophila* pupae expressing *LOV-LexA* and *CD8:GFP* in oenocytes were mounted on double-side sticky tape, and an opening in the pupal case that exposes oenocytes was created. Pupae expressed *LOV-LexA* and *CD8:GFP* in oenocytes with the following genotype: *w; 109(2)-GAL4, UAS-CD8:GFP/+; UAS-LOV-LexA/+*. l) Representative images of pupal oenocytes showing LOV-LexA before (before) and immediately following exposure to blue light (after), 60 min after exposure to blue light (recovery), and 120 min after light exposure (final scan). The final scan included the green channel to capture *CD8:GFP*, coexpressed with *LOV-LexA*, and used to delineate the cell body. The light used to capture GFP is blue and elicited another translocation of LOV-LexA to the nucleus, thereby demonstrating that the oenocytes were healthy after imaging. m) Mean nuclear tdTomato fluorescence over time, imaged live every 5 min. Shades represent standard error of the mean (SEM). LOV-LexA translocates to the nucleus upon exposure to blue light within minutes in oenocytes and slowly leaks out of the nucleus after exposure to blue light.

*Drosophila* larvae have transparent cuticle that allows for internal tissues to be exposed to unabated light. The bilateral, multilobed fat body running along the larva, is visible underneath the body wall musculature and is targeted by the collagenase enhancer (*Cg-*)*GAL4* (Asha *et al.* 2003). Distribution of eLOV-nls-tdTomato-mLexA:GAD, eLOV-nls-tdTomato-mLexA:p65, or eLOV-nls-tdTomato-mLexA:VP16 in larval fat body followed the trend observed in S2R+ cells (Fig. 2, a and d and Supplementary Fig. 2, a–f), with eLOV-nls-tdTomato-mLexA:GAD forming clusters (Supplementary Fig. 2, c and d) and eLOV-nls-tdTomato-mLexA:p65 or eLOV-nls-tdTomato-mLexA:VP16 distributing evenly in the cytoplasm, and occasionally in the nucleoplasm (Fig. 2, c, d, f, and g and Supplementary Fig. 2, f and g). To test the ability to induce expression of a reporter under control of LexAop sequences, *LexAop-CsChrimson:Venus* (Klapoetke *et al.* 2014) (hereafter referred to as *Venus*), second and third instar larvae reared at 18°C were placed in 96-well plates in a 15% sucrose solution, to repress their tendency to wander, and exposed to several pulses of low intensity blue light (Fig. 2, a and b and Supplementary Table 3). Larvae were then incubated at 25°C for 7–11 h, after which the fat body was dissected, fixed, and stained. Despite considerable expression of *eLOV-nls-tdTomato-mLexA:GAD* and *eLOV-nls-tdTomato-mLexA:VP16* in fat body cells, exposure to blue light failed to elicit expression of the reporter (Supplementary Fig. 2, a–h). In contrast, exposure of larvae expressing *eLOV-nls-tdTomato-mLexA:p65* to as little as three pulses of blue light led to increase in reporter expression under control of LexAop sequences (Fig. 2a–i). Surprisingly, a lower number of pulses of blue light combined with longer incubation at 25 °C resulted in maximum increase in reporter expression (Fig. 2i). This suggests that despite low intensity, exposure to too many pulses of blue light leads to less efficiency of light-gated expression. Given that expression of *eLOV-nls-tdTomato-mLexA:p65* in S2R+ and fat body cells kept in the dark presented no or very low expression of the reporter gene and that exposure to blue light led to increase in reporter expression, the construct *eLOV-nls-tdTomato-mLexA:p65* was selected for further studies and named *LOV-LexA* (Fig. 1g).

The use of the AsLOV2 domain to cage a NLS signal has been previously demonstrated to effectively move coupled proteins into the nucleus in a light-dependent manner (Niopek *et al.* 2014; Yumerefendi *et al.* 2015). To determine the kinetics of LOV-LexA nuclear translocation, we expressed *LOV-LexA* in oenocytes, which are large cells sitting underneath the cuticle with roles in secretion and metabolism (Makki *et al.* 2014). Adult oenocytes arise in pupae and reach their final locations through several bouts of migration during metamorphosis. We imaged stationary oenocytes in pupae aged between 2 and 3 days APF, through the transparent cuticle, after removal of the overlying pupal case (Fig. 2, j and k). After preparation of the samples, LOV-LexA was present in the cytoplasm as well as in the nucleus in abdominal oenocytes (Fig. 2l “before”). Exposure to blue light (485 nm, 2.53 µW, 30 slices, Supplementary Table 3) leads to a rapid accumulation of LOV-LexA in the nucleus, that decreases over time (Fig. 2l “after,” “recovery,” Fig. 2m). To determine the location of the cytoplasm, cells were imaged to detect CD8:GFP as well as tdTomato in LOV-LexA 100 min after exposure to blue light (Fig. 2l “final scan,” not depicted in the graph in Fig. 2m). LOV-LexA accumulated again in the nucleus in all oenocytes imaged (*n* = 14), indicating that the reduction of LOV-LexA levels in the nucleus over time is not due to the general degradation of the live preparation. LOV-LexA thus exhibits fast translocation to the nucleus, which peaks 5 min after exposure to blue light, and a slower movement out of the nucleus, reaching minimum levels after 20 min in the dark.

### LOV-LexA behavior in diverse neuronal types

Similar to fat body, we assessed LOV-LexA behavior in neurons with the transgene Venus under control of LexAop sequences (*LexAop-CsChrimson:Venus*) (Klapoetke *et al.* 2014) as a reporter of LOV-LexA transcriptional activity. Presence of CsChrimson:Venus is readily detected by its native fluorescence in neurons with fixation alone, thereby eliminating the need for the extra amplification step of antibody immunostaining (McKellar *et al.* 2019). We tested LOV-LexA in the lobula columnar 10 (LC10)-group neurons, LC10a, b, c, and d, that arborize in the lobula and project to anterior optic tubercle, in the dorsal fly brain (Otsuna and Ito 2006; Costa *et al.* 2016; Panser *et al.* 2016; Wu *et al.* 2016). LC10a neurons, but not LC10b, c, or d, mediate tracking of visual objects (Ribeiro *et al.* 2018; Hindmarsh Sten *et al.* 2021). Expression of LOV-LexA in LC10-group neurons with *LC10s-SS2* and *LC10a-SS1* drivers (Ribeiro *et al.* 2018) led to moderate expression of Venus in the dark if flies were raised at 25°C (Supplementary Fig. 3, a–e), but not if flies were raised at 18°C in the dark (Supplementary Fig. 3, f–j). This indicates that the dark state of LOV-LexA is unstable in flies reared at 25°C. The leakiness of LOV-LexA at 25°C could arise from an elevated accessibility to the NLS at higher temperatures or increased LOV-LexA expression as previously observed in S2R+ cells (Supplementary Fig. 1) and in other eLOV-based tools (Kim, Wang, *et al.* 2017; Kim *et al.* 2019). The stability of LOV-LexA in the dark was further tested with the panneuronal driver *GMR57C10-GAL4* (Jenett *et al.* 2012). In many neuronal types, rearing flies at 18°C prevented the accumulation of the Venus reporter in flies expressing LOV-LexA panneuronally (Supplementary Fig. 3, k–m). Several neuronal types, including neurons in the optic lobe, mushroom body, antennal lobe, and suboesophageal region, were an exception to this rule and presented high levels of *Venus* expression. Differences in expression strength across neuronal types represented in the *GMR57C10-GAL4* expression pattern partially account for the observed variability in *Venus* expression in the dark. On the other hand, differential expression pattern of genes involved in nucleocytoplasmic transport in different neuronal types could potentially underlie these discrepancies. Alpha importins function as adaptors that bind NLS peptides, bringing proteins with NLS in contact with β importins, which in turn mediate transport into the nucleus. The ɑ importin *ɑ Karyopherin 4* (*ɑKap4*, *CG10478*) is highly expressed in Kenyon cells and other neuronal types (Supplementary Fig. 3n) (Venken *et al.* 2011; Larkin *et al.* 2021). Expression of the ɑ importin *karyopherin ɑ1* (*Kap-ɑ1*, *CG8548*) and the β importins *cadmus* (*cdm*, CG7212) and *Chromosome segregation 1* (*Cse1*, *CG13281*) are limited to a small number of neuronal types in the central brain (Supplementary Fig. 3, o–q). The presence of *ɑKap4*, or other importins, in certain neuronal types could potentially explain the selected leakiness of LOV-LexA dark state. To test this, we coexpressed *Kap-ɑ1* (Jang *et al.* 2015; Larkin *et al.* 2021) with *LOV-LexA* in *LC10a-SS1* neurons in flies reared at 18°C in the dark. Coexpression of *LOV-LexA* with *Kap-ɑ1* in *LC10a-SS1* neurons led to the expression of Venus reporter gene (Supplementary Fig. 3, r–v), suggesting that increase in nucleocytoplasmic transport may facilitate the translocation of LOV-LexA to the nucleus, in the dark.

We tested several GAL4 and split-GAL4 drivers in flies raised at 18°C and compared the expression of *LOV-LexA* and the reporter *Venus*. Like in other cell types, above certain levels of expression of *LOV-LexA*, the amount of *Venus* detected in neurons correlated with that of *LOV-LexA* (Supplementary Fig. 3z). Together these observations suggest that the LOV-LexA tool has a stable dark state in drivers of weak to moderate expression strength, which constitute the majority of GAL4 and split-GAL4 lines available for genetic access to single neuronal types.

### LOV-LexA mediates light-gated expression in neurons

**Fig. 3.**
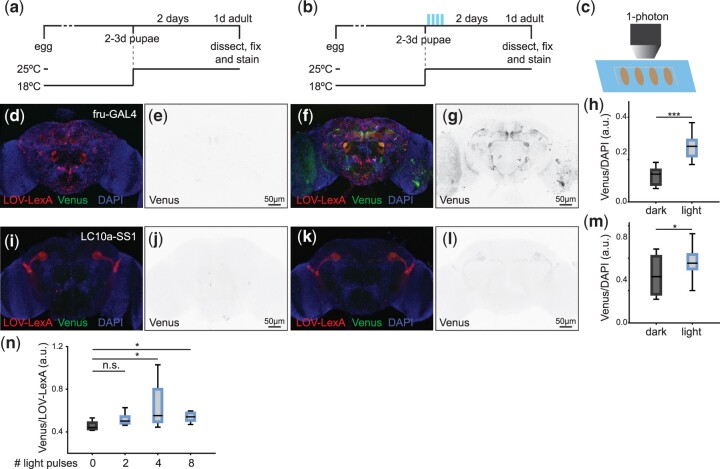
LOV-LexA gates expression with light in neurons. a, b) Schematic representation outlining the experiment. Pupae reared at 18°C aged 2–3 days APF were removed from vials, mounted on double side sticky tape on a cover slip and kept in the dark (a), or pasted onto a slide and exposed to blue light (b). Mounted pupae kept in the dark or exposed to light were shifted to 25°C until dissection. c) Schematic representation showing pupae lined on double side sticky tape for light delivery. Adult brains showing expression of *LOV-LexA* (red in d and f) and *LexAop-CsChrimson:Venus* (*Venus* in d and f and dedicated image in e and g), as detected by native fluorescence of tdTomato and Venus, from pupae kept in the dark (d, e) or exposed to light (f, g) at 3–4 days APF, as shown in (b). h) Ratio of Venus signal intensity over DAPI signal intensity for *fru+* neuronal cell bodies located in the anterior brain. Pupae exposed to pulses of blue light (N=12) express the LexAop reporter *Venus* at higher levels compared to pupae kept in the dark (*N* = 13), demonstrating that exposure to light leads to higher LOV-LexA transcriptional activity. Adult brains showing expression of *LOV-LexA* (red in i and k) and *LexAop-CsChrimson:Venus* (*Venus*, green in i and k and dedicated image in j and l), as detected by the native fluorescence of tdTomato and Venus, from *w, LexAop-CsChrimson:Venus;+/LC10a-SS1.AD; UAS-LOV-LexA/LC10a-SS1.DBD* pupae kept in the dark (i, j) or exposed to light at 3–4 days APF (k, l), as shown in (b). m) Ratio of Venus signal intensity over DAPI signal intensity for LC10a neuronal cell bodies. Pupae exposed to pulses of blue light (*N* = 12) express the LexAop reporter *Venus* at higher levels compared to pupae kept in the dark (*N* = 6). n) Ratio of Venus over LOV-LexA native fluorescence from adult brains *w, LexAop-CsChrimson:Venus;+; UAS-LOV-LexA/fru-GAL4* exposed to 0, 2, 4, or 8 pulses of blue light as 2–3 days APF pupae (*N* = 3, 4, 11, and 5, respectively). *** represents *P*-values <0.001, * represents *P*-values <0.05, n.s. represents *P*-values >0.05, 2-tailed Mann–Whitney tests.

The pupal case and the adult cuticle are tanned and block light, leading to the decreased exposure of internal tissues to light. To uncage the NLS in LOV-LexA expressed in pupal and adult brain, we used a 1-photon laser with 458-nm wavelength in a confocal microscope (see *Materials and Methods* and Supplementary Table 3). The driver *fru-GAL4*, a *GAL4* knock-in in the locus of the gene *fruitless* (*fru*) (Gailey and Hall 1989; Stockinger *et al.* 2005), targets approximately 100 neuronal types, collectively called *fru* neurons, many of which were shown to regulate courtship behavior (among others, Cachero *et al.* 2010; Yu *et al.* 2010; Lu *et al.* 2012; Thistle *et al.* 2012; Toda *et al.* 2012; Bath *et al.* 2014; Inagaki *et al.* 2014; Ribeiro *et al.* 2018; McKellar *et al.* 2019). Expression is initiated in pupal development with low expression strength at late pupal stages. Pupae reared at 18°C and expressing *LOV-LexA* in *fru* neurons were exposed to a series of 4 preprogrammed light pulses (Supplementary Table 3), after which they were placed at 25°C for 2 days (Fig. 3, a–c). Expression of Venus was significantly increased in most *fru* neurons in pupae that were exposed to blue light (Fig. 3, d–h). Importantly, pupae kept in the dark displayed little or no expression of *Venus* (Fig. 3, d and e). Similar outcomes were observed for the *LC10a-SS1* driver. Like *fru-GAL4*, *LC10a-SS1* drives expression in pupal stages at low levels (Ribeiro *et al.* 2018, and data not shown). Exposure of pupae to 4 pulses of 1-photon laser 458 nm light spaced over 30 min (Supplementary Table 3) elicited light-dependent expression of *Venus* in LC10a neurons (Fig. 3, i–m). Delivery of 4–8 pulses of blue light, but not 2, proved to be sufficient for appreciable increase in *Venus* expression (Fig. 3n). Increase in expression of the reporter *Venus* in *fru+* and LC10-group neurons exposed to light, and its absence in the same neurons kept in the dark, demonstrates that LOV-LexA gates expression with blue light in neurons in the pupal brain.

**Fig. 4.**
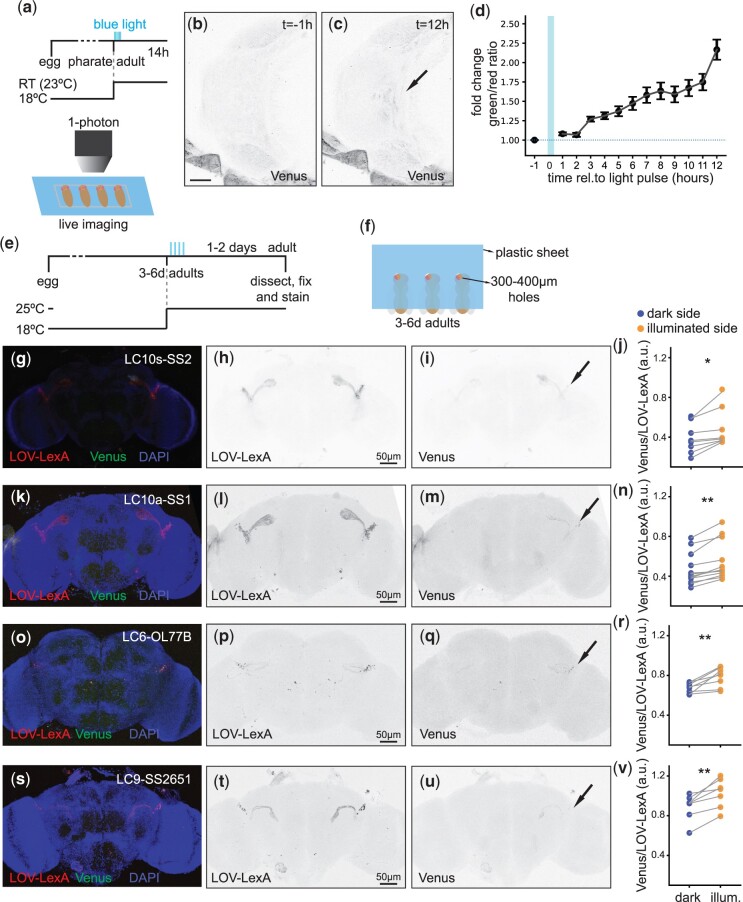
LOV-LexA enables spatial and temporal control of transgene expression with light. a) Schematic representation outlining the experiment (top) and schematic representation showing pupae lined up on a slide, with exposed heads for live imaging and blue light delivery (bottom), shown in (c) and (d). Live image of 4 day APF pupal head after removal of the pupal case, with expression of *Venus* in *fru+* neurons (*w, LexAop-CsChrimson:Venus;+; UAS-LOV-LexA/fru-GAL4*) before delivery of blue light (b) and 12 h after delivery of blue light (c). d) Change in the ratio of native Venus signal over LOV-LexA tdTomato native signal, before and after light delivery (*N* = 5). e) Timeline of the experiment. f) Schematic representation showing preparation to deliver spatially restricted light to immobilized adult flies, glued with low temperature melting wax to an opaque coverslip, with the head placed under a hole with a diameter between 300 and 400 µm. g-i, k-m, o-q) Representative images of adult brains expressing *LOV-LexA* in several LC neurons and spatially restricted LexAop-CsChrimson:Venus, after exposure to spatially restricted blue light to target visual projection neurons unilaterally and quantification. g–j) LC10-group neurons *LC10s-SS2* (*w, LexAop-CsChrimson:Venus; +/LC10s-SS2.AD; UAS-LOV-LexA/LC10s-SS2.DBD*) with *N* = 8. k–n) LC10a neurons *LC10a-SS1* (*w, LexAop-CsChrimson:Venus; +/LC10a-SS1.AD; UAS-LOV-LexA/LC10a-SS1.DBD*) with *N* = 11. o–r) *LC6-OL77B* neurons (*w, LexAop-CsChrimson:Venus; +/OL77B.AD; UAS-LOV-LexA/OL77B.DBD*) with *N* = 8. s–v) *LC9-SS2651* neurons (*w, LexAop-CsChrimson:Venus; +/SS2651.AD; UAS-LOV-LexA/SS2651.DBD*) with *N* = 7. j, n, r, v) Ratio of native Venus over native LOV-LexA (tdTomato) signals between the side of the head that was illuminated compared to the side that was kept in the dark, with each plot corresponding to the genotypes shown in the same row. * represents *P*-values <0.05, ** represents *P*-values <0.01, Wilcoxon test.

Precise control of the time of initiation of transgene expression has numerous advantages, including allowing for embryonic and pupal development to occur undisturbed in the absence of ectopic expression and for regulation of the level of transgene expressed. We measured the time it takes for LOV-LexA to drive the transcription of LexAop-controlled *Venus* after exposure to blue light. The head in pupae expressing *LOV-LexA* with *fru-GAL4* was uncovered by removing the encapsulating pupal case and exposed to pulses of blue light (Supplementary Table 3 and Fig. 4a). The pupal brain was then imaged every hour for 12 h to determine the timing at which *Venus* starts to be expressed. *Venus* expression doubled 12 h after blue light pulse delivery (Fig. 4, b–d). Detection of expression with native protein fluorescence in adult brains was reliably observed 24 h after exposure to blue light during late pupal stages (Fig. 3, d–h), indicating that LOV-LexA light-gated expression takes 12–24 h to accumulate enough LexAop Venus reporter to be visualized with native levels.

Drivers that initiate expression at adult stages, such as *LC10s-SS2*, were exposed to light at adult stages. Adult flies expressing *LOV-LexA* in LC10-group neurons were immobilized with low-melting wax on a custom-made opaque plastic coverslip with 300–400-µm holes (Fig. 4, e and f). The area of cuticle above the cells of interest was placed under one of the holes (Fig. 4f). Somata for the LC10-group neurons are located in the dorso-posterior side of the head, in an area bordering the rim of the retina. Immobilized flies with the cuticle covering somata of LC10-group neurons on one side of the adult head exposed were delivered 4–6 pulses of 485 nm 1-photon laser light over the course of 1 h (Supplementary Table 3). Detection of native fluorescence revealed the accumulation of Venus in LC10-group neurons exclusively on the side exposed to light (Fig. 4, g–j). Similar light deliveries to adult flies expressing *LOV-LexA* in LC10a (Fig. 4, k–n), LC6 (Fig. 4, o–r), and LC9 neurons (Fig. 4, s–v) resulted in unilateral *Venus* expression. Importantly, most flies prepared in this fashion showed unilateral expression in LC neurons (Fig. 4, j, n, r, and v), indicating that LOV-LexA allows for consistent genetic access to the same cell types within an expression pattern.

## Discussion

We developed LOV-LexA, a light-gated expression system based on the photosensitive eLOV domain (Wang *et al.* 2017), and the modified transcription factor mLexA (Emelyanov and Parinov 2008; Pfeiffer *et al.* 2010). In the absence of light, LOV-LexA proteins reside in the cytoplasm of larval and adult cells. Delivery of blue light causes the LOV Jɑ helix to uncage an NLS, which then mediates translocation of LOV-LexA to the nucleus. Once in the nucleus, LOV-LexA drives expression of transgenes under the control of LexAop sequences. The use of light as a trigger enables control of expression with high spatial and temporal resolution in live larvae and adult flies, making LOV-LexA an important addition to the *Drosophila* genetic toolbox that will expand the use of existent broad drivers as well as allow targeting subsets of cells within single tissues or cell types.

Several forms of LexA-transactivator chimeras are used in different animal models (Lai and Lee 2006; Emelyanov and Parinov 2008; Nonet 2020). Surprisingly, the ability to remain outside the nucleus in the dark and to elicit reporter expression upon light exposure varied widely among different combinations of mLexA-transactivator chimeras, eLOV-nls, and fluorescent tag. Replacing tdTomato with the FLAG tag in LOV-LexA, to make eLOV-nls-FLAG-mLexA:p65, leads to high levels of leakiness in the dark in S2R+ cells (data not shown), suggesting that intraprotein interactions among the different components of LOV-LexA play an important role in stability of the Jɑ helix in the dark (Kim, Wang, *et al.* 2017; Wang *et al.* 2017). Experiments in cell culture suggest that at high levels of expression, LOV-LexA proteins are more likely to translocate to the nucleus and drive expression of the LexAop reporter transgene. Rearing flies expressing LOV-LexA at 25°C similarly leads to unwanted expression of the LexAop reporter, imposing limits on the temperature used to raise fruit flies and the available driver lines. Further improvements of the eLOV domain have to be implemented to circumvent this limitation (Kim *et al.* 2019). Roughly 12–24 h separate the delivery of blue light and the accumulation of LexAop transgene expression in neurons, giving the fly time to recover from potential adverse effects of exposure to blue light, that include temporary blindness (Montell 2012). This temporal separation might preclude the use of transgenes encoding proteins with a short half-life. However, this time allows for other light and genetic manipulations to be performed on the same animal, without the need to perform all manipulations simultaneously on a tethered fly (Kim, Rouault, *et al.* 2017).

Replacing the transcription factor in LOV-LexA with QF2 (Riabinina and Potter 2016), testing NLS sequences of varied strengths, or using other LOV-based domains might improve the stability of LOV-LexA at higher temperatures and expression levels and change the time required for reporter expression. Addition of another protein domain that counterbalances nuclear import, such as a nuclear export signal (Niopek *et al.* 2014) or a membrane tethering domain (Kim, Wang, *et al.* 2017), might provide more stability to LOV-LexA. On the other hand, some neuronal types present LexAop reporter expression even if *LOV-LexA* is expressed at low levels. The uneven expression of importins across the fly brain, similar to what is observed in the mouse brain (Hosokawa *et al.* 2008), suggests that different neuronal types might express nucleocytoplasmic transport machinery to different extents. We predict that this variability is likely to influence how LOV-LexA functions across neuronal and cell types, making cells with high nucleocytoplasmic transport capabilities less suitable for light-gated expression with LOV-LexA.

Other light-gated expression systems have been developed in *Drosophila*, including the cryptochrome split-LexA, Photo-Gal4, and ShineGal4 (Chan *et al.* 2015; de Mena and Rincon-Limas 2020; di Pietro *et al.* 2021). We expressed the cryptochrome split-LexA with the same driver used to test LOV-LexA, *LC10s-SS2* (Ribeiro *et al.* 2018), and found that cryptochrome split-LexA system is leaky in flies raised at 18°C and kept in the dark (Supplementary Fig. 3, x and y). Given that Photo-GAL4 relies on PhyB and requires addition of the chromophore PCB, normally absent in animal cells, it is currently limited to *ex vivo* studies (de Mena and Rincon-Limas 2020). The chromophore providing LOV with light sensitivity, flavin, exists in animal cells, making the LOV-LexA system solely dependent on the delivery of light. The limited number of enhancers driving ShineGal4, mostly targeting embryonic and pupal epithelia, prevents its widespread testing without re-cloning under other promoters. LOV-LexA is currently under the control of the UAS promoter and is one cross away from being tested with the myriad of GAL4 and split-GAL4 driver lines available.

There are thousands of enhancer-LexA or -GAL4 drivers targeting several cell types simultaneously (Jenett *et al.* 2012; Kockel *et al.* 2016; Robie *et al.* 2017; Tirian and Dickson 2017; Kockel *et al.* 2019). The LOV-LexA can be placed downstream of broadly expressed enhancers, to restrict transgene expression in the cell type of interest. Moreover, LOV-LexA downstream of an enhancer can be combined with GAL4 and QF binary expression systems, to genetically target two or more single neuronal types independently in the same animal, enabling several different experiments, including simultaneous monitoring of neuronal activity or determining dependency relationships among different neuronal types. Many neuronal types are composed of dozens of cells that are topographically organized to represent the visual field (Fischbach and Dittrich 1989; Otsuna and Ito 2006; Wu *et al.* 2016). Topographic organization of neuropiles processing sensory information is also observed in other animals, like the mouse superior colliculus, visual cortex, and for other sensory modalities (Engelmann *et al.* 2021; Huberman *et al.* 2008; Nassi and Callaway 2009; Petersen 2019). LOV-LexA is an ideal tool to test the role of topography, by providing consistent genetic access to the same subsets of somata within a single neuronal type, with little stochasticity. We demonstrate consistent targeting of several LC neurons unilaterally with LOV-LexA by targeting their somata. Applying this strategy to all visual projection neurons will elucidate how each contributes to guiding visual behavior.

Compared to *D. melanogaster*, many model organisms in which it is possible to create transgenics have smaller repertoires of enhancer driver lines that give access to different tissues and cell types. Implementing LOV-LexA in such model organisms will greatly amplify the number of specific cell types that can be genetically manipulated, expanding the landscape of possible experiments in emerging model organisms and the knowledge we can acquire from them.

## Supplementary Material

jkac178_Supplementary_Data

## Data Availability

The DNA plasmid for LOV-LexA is deposited in DGRC (stock # 1583) and is available upon request. The *D. melanogaster* LOV-LexA flies were deposited in VDRC (stock # 311200) and are also available upon request. Data sets are available upon request. Supplemental material is available at *G3* online.
