# Supplementary material for: Spatial and temporal control of expression with light-gated LOV-LexA
Source: G3 (Bethesda). 2022 Jul 25;12(10):jkac178. doi: 10.1093/g3journal/jkac178 (PMC9526042; doi:10.1093/g3journal/jkac178)
Supplement: jkac178_Supplementary_Data [file jkac178_supplementary_data.zip › Ribeiroetal_ms_supplemental_front_page_4MB.pdf]

G3 Genes|Genomes|Genetics

Supplemental material

Spatial and temporal control of expression with light-gated LOV-LexA

Inês M.A. Ribeiro, Wolfgang Eßbauer, Romina Kutlesa, and Alexander Borst

Supplementary Figure 1

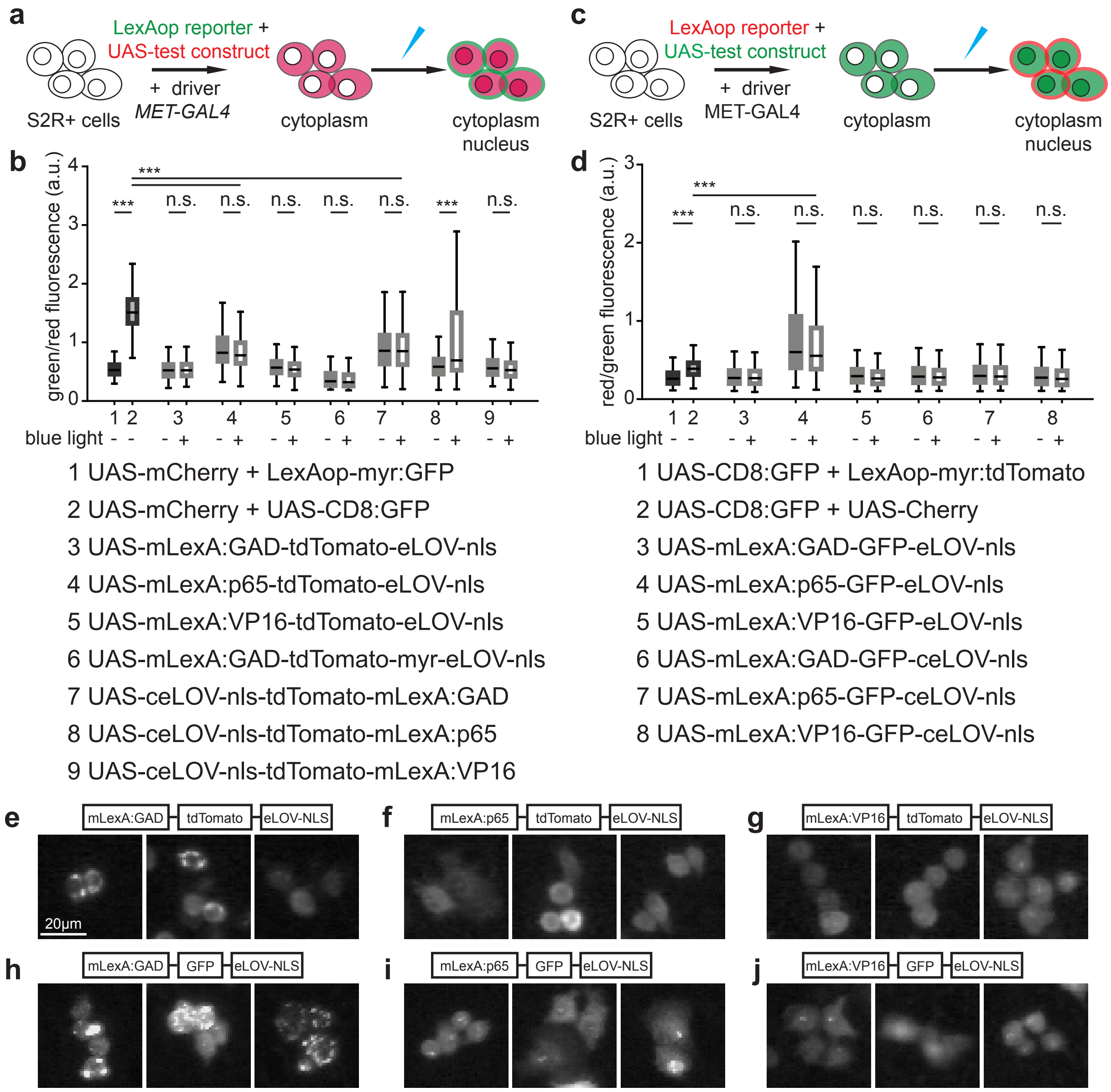

Supplementary Fig. 1: Design of a light-gated expression system based on LOV (suppl. to Figure 1).

A. S2R+ cells were transfected with the driver pMET-GAL4, the LexAop reporter LexAop-myr:GFP and the test construct. Cells were then kept in the dark or exposed to light. **B.** Ratio of LexAop reporter myr:GFP expression in relation to expression of the different test constructs determined by tdTomato fluorescence. As in Figure 1B, co-transfection of UAS-mCherry with the LexAop reporter LexAop-myr:GFP established the baseline (first bar in the boxplot), whereas co-transfection of UAS-mCherry and UAS-CD8:GFP was used as an approximate measure of co-expression (second bar in the boxplot). Only construct 8, ceLOV-nls-tdTomato-mLexA:p65 (codon optimized ceLOV), elicits expression of the reporter upon light exposure. The tests constructs are indicated below the boxplot; ceLOV stands for eLOV codon optimized for *Drosophila*. At least 200 cells with medium levels of expression of mCherry or tdTomato, from at 2 to 5 transfections of S2R+ cells are represented for each condition. *** represents p values < 0.001, n.s. represents p values > 0.05, obtained with Student's t test. **C.** S2R+ cells were transfected with a red reporter, LexAop-myr:Tomato, together with test constructs bearing GFP as a tag for visualization. **D.** Ratio of LexAop reporter myr:tdTomato expression in relation to expression of test constructs tagged with GFP. As above, co-transfection of UAS-mCherry and UAS-CD8:GFP served as an approximate measure of co-expression. Co-transfection of UAS-CD8:GFP with the LexAop-myr:tdTomato indicated baseline expression levels. At least 200 cells with medium levels of expression of mCherry or tdTomato, from at 2 to 5 transfections of S2R+ cells are represented for each condition. *** represents p values < 0.001, n.s. represents p values > 0.05, obtained with Student's t test. **E-J.** Representative examples of S2R+ cells expressing mLexA:GAD-tdTomato-eLOV-nls (E), mLexA:p65-tdTomato-eLOV-nls (F), mLexA:VP16-tdTomato-eLOV-nls (G), mLexA:GAD-GFP-eLOV-nls (H), mLexA:p65-GFP-eLOV-nls (I), mLexA:VP16-GFP-eLOV-nls (J) showing subcellular distribution of each of these combinations. All combinations bearing LexA:GAD form clusters in the cytoplasm, whereas combinations bearing LexA:p65 or LexA:VP16 are more likely to be distributed evenly in the cytoplasm, and sometimes the nucleoplasm.

Supplementary Figure 2

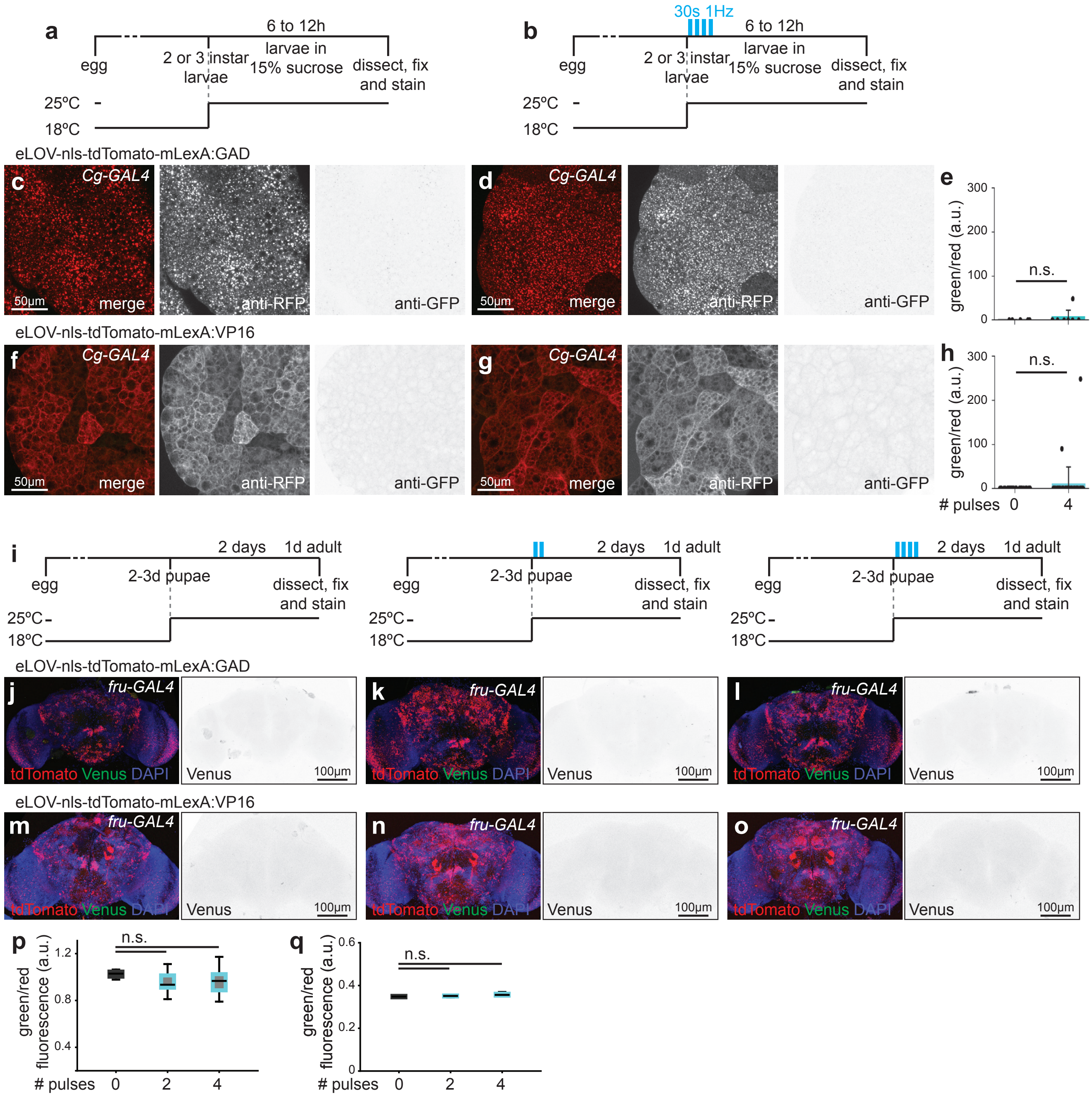

Supplementary Fig. 2: LexA chimeras LexA:GAD and LexA:VP16 combined with N-terminal eLOV are unable to elicit expression of LexAop reporter (suppl. To Figure 2).

A,B. Schematic representing the timeline of fly rearing temperature and light delivery for the fat bodies in C to H. **C-H.** Fat bodies of second to third instar larvae expressing eLOV-nls-tdTomato-mLexA:GAD (*w, LexAop-CsChrimson:Venus; Cg-GAL4/+; UAS-eLOV-nls-tdTomato-mLexA:GAD/+* C-E) or eLOV-nls-tdTomato-mLexA:VP16 (*w, LexAop-CsChrimson:Venus; Cg-GAL4/+; UAS-eLOV-nls-tdTomato-mLexA:VP16/+* F-H), kept in the dark (C, F) or exposed to four pulses of blue light (each pulse lasting 30s at 1Hz), and incubated 12h at 25ºC. The ratio of pixel intensity of anti-GFP signal (LexAop reporter)/anti-RFP signal (test construct) for stained fat bodies is shown for eLOV-nls-tdTomato-mLexA:GAD in E (dark N=3, light N=6) and eLOV-nls-tdTomato-mLexA:VP16 in H (dark N=3, light N=4); n.s. represents p value > 0.05, obtained with Student's t test. **I.** Schematic representing the timeline of fly rearing temperature and light delivery for the brains in J to Q. **J-Q.** Adult brains showing expression of LOV-LexA (red) and LexAop-CsChrimson:Venus (Venus, green in merge, and isolated dedicated image on the right) from *w, LexAop-CsChrimson:Venus;+;UAS--eLOV-nls-tdTomato-mLexA:GAD/fru-GAL4* (J-L, P) and *w, LexAop-CsChrimson:Venus;+;UAS--eLOV-nls-tdTomato-mLexA:VP16/fru-GAL4* (M-O, Q) pupae kept in the dark (J, M) or exposed to light (K,L, N, O) at 3-4 days APF, as shown in I. **P,Q.** Ratio of native green (Venus) and red (tdTomato in test construct) fluorescence for cell bodies. Different protocols for light delivery failed to elicit Venus expression in brains expressing eLOV-nls-tdTomato-mLexA:GAD (dark N=3, 2 light pulses N=10, 4 light pulses N=7) or eLOV-nls-tdTomato-mLexA:VP16 (dark N=2, 2 light pulses N=5, 4 light pulses N=4) under control of *fru-GAL4*; n.s. represents p value > 0.05, obtained with Student's t test.

Supplementary Figure 3

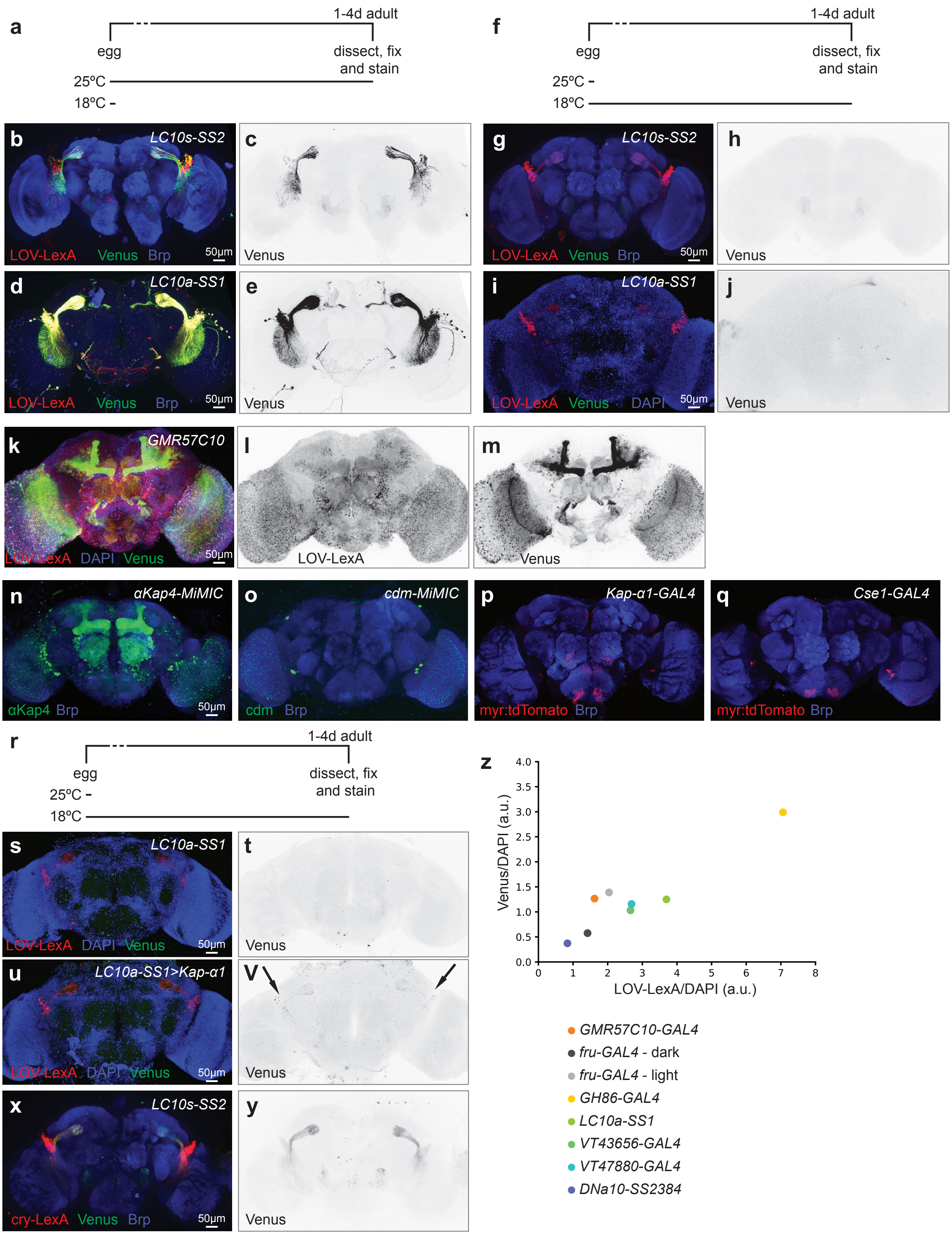

Supplementary Fig. 3: LOV-LexA tests in neurons.

A. Schematic representing the timeline of fly rearing for the brains shown in B to E. **B-E.** LOV-LexA under control of *LC10s-SS2* (B, N=4) or *LC10a-SS1* (D, N=4) driver in flies reared at 25ºC shows expression of the LexAop-reporter transgene Venus (C, E). **F.** Schematic representing the timeline of fly rearing for the brains shown in G to J. **G-J.** LOV-LexA under control of *LC10s-SS2* (G, N=5) or *LC10a-SS1* (I, N=2) driver in flies reared at 18ºC shows no expression of the LexAop-reporter transgene Venus (H, J). **K-M.** LOV-LexA (K, L) under control of a panneuronal driver GMR57C10-GAL4 showing LOV-LexA distribution predominantly in cell bodies (L) and uncorrelated expression of the LexAop-reporter Venus (M) in different neurons, N=6. **N, O.** Expression of the EGFP-tagged importins *aKap4* and *cdm* with MiMIC lines $aKap4^{MI0631}$ (N=6), and $cdm^{MI06239}$ (N=4). **P, Q.** Expression pattern of importins *Kap-a1* and *Cse1* visualized with myr:tdTomato under control of GAL4 inserted into *Kap-a1* (N=4) and *Cse1* (N=5) gene loci. **R.** Schematic representing the timeline of fly rearing for the brains shown in S to Y. **S-V.** Ectopic expression of *Kap-a1* in flies reared at 18ºC and kept in the dark renders LOV-LexA leaky (U, V with N=3) compared to expression of LOV-LexA alone under the same conditions (S, T, with N=5). **X-Y.** Flies expressing cryptochrome split-LexA reared at 18ºC in the dark present expression of the LexAop reporter Venus. **Z.** Average ratio of Venus native signal intensity relative to DAPI signal intensity in relation to the average ratio of tdTomato native signal intensity relative to DAPI signal intensity for flies raised at 18ºC and kept in the dark (except for fru-GAL4 – light), to test the dark state of LOV-LexA for various drivers of different strength shows that above certain levels of expression, Venus expression correlates with LOV-LexA expression level, even in the absence of light exposure. If expressed at moderate to low levels, LOV-LexA maintains low transcriptional activity, that increases with light exposure, as is the case for *fru-GAL4* (N=12 exposed to light, N=13 kept in the dark, see Figure 3 D-H). For other drivers, N=2 to 5 brains from flies raised in the dark at 18ºC, are plotted.

Supplementary Table S1. Genetic constructs used in this study.

Name	Features	Vector	Promoter	Details
c204	tdTomato-mLexA:GAD	pJFRC7	UAS	modified LexA:GAD codon optimized for *Drosophila*
c205	tdTomato-LexA:GAD	pJFRC7	UAS	LexA:GAD codon optimized for *Drosophila*
c214	tdTomato-mLexA:p65	pJFRC7	UAS	Modified LexA:p65 codon optimized for *Drosophila*
c215	tdTomato-LexA:p65	pJFRC7	UAS	LexA:p65 codon optimized for *Drosophila*
c224	tdTomato-mLexA:VP16	pJFRC7	UAS	Modified LexA:VP16 codon optimized for *Drosophila*
c225	tdTomato-LexA:VP16	pJFRC7	UAS	LexA:VP16 codon optimized for *Drosophila*
c11	eLOV-nls-tdTomato-mLexA:GAD	pJFRC7	UAS	LexA:GAD codon optimized for *Drosophila*, with NLS-like modified
c12	eLOV-nls-tdTomato-mLexA:p65	pJFRC7	UAS	LexA:p65 codon optimized for *Drosophila*, with NLS-like modified
c13	eLOV-nls-tdTomato-mLexA:VP16	pJFRC7	UAS	LexA:VP16 codon optimized for *Drosophila*, with NLS-like modified
c17	mLexA:GAD-tdTomato-eLOV-nls	pJFRC7	UAS	LexA:GAD codon optimized for *Drosophila*, with NLS-like modified
c18	mLexA:p65-tdTomato-eLOV-nls	pJFRC7	UAS	LexA:p65 codon optimized for *Drosophila*, with NLS-like modified
c19	mLexA:VP16-tdTomato-eLOV-nls	pJFRC7	UAS	LexA:VP16 codon optimized for *Drosophila*, with NLS-like modified
c111	ceLOV-nls-tdTomato-mLexA:GAD	pJFRC7	UAS	LexA:GAD codon optimized for *Drosophila*, with NLS-like modified<br>eLOV evolved LOV codon optimized for *Drosophila*
c121	ceLOV-nls-tdTomato-mLexA:p65	pJFRC7	UAS	LexA:p65 codon optimized for *Drosophila*, with NLS-like modified<br>eLOV evolved LOV codon optimized for *Drosophila*
c131	ceLOV-nls-tdTomato-mLexA:VP16	pJFRC7	UAS	LexA:VP16 codon optimized for *Drosophila*, with NLS-like modified<br>eLOV evolved LOV codon optimized for *Drosophila*

Name	Features	Vector	Promoter	Details
c26	mLexA:GAD-GFP-eLOV-nls	pJFRC7	UAS	LexA:GAD codon optimized for *Drosophila*, with NLS-like modified
c27	mLexA:p65-GFP-eLOV-nls	pJFRC7	UAS	LexA:p65 codon optimized for *Drosophila*, with NLS-like modified
c28	mLexA:VP16-GFP-eLOV-nls	pJFRC7	UAS	LexA:VP16 codon optimized for *Drosophila*, with NLS-like modified
c29	mLexA:GAD-GFP-ceLOV-nls	pJFRC7	UAS	LexA:GAD codon optimized for *Drosophila*, with NLS-like modified<br>eLOV evolved LOV codon optimized for *Drosophila*
c30	mLexA:p65-GFP-ceLOV-nls	pJFRC7	UAS	LexA:p65 codon optimized for *Drosophila*, with NLS-like modified<br>eLOV evolved LOV codon optimized for *Drosophila*
c31	mLexA:VP16-GFP-ceLOV-nls	pJFRC7	UAS	LexA:VP16 codon optimized for *Drosophila*, with NLS-like modified<br>eLOV evolved LOV codon optimized for *Drosophila*
myrTom	LexAop-myr:tdTomato	pJFRC19	LexAop	LexAop driving tdTomato expression; reporter for activity of LexA-transactivator-eLOV-tag with GFP or FLAG
mCherry	mCherry	pJFRC7	UAS	UAS driving mCherry expression; reporter for transfection efficiency and negative control for LexA-transactivator-eLOV-tag constructs
myrGFP	LexAop-myr:GFP	pJFRC19	LexAop	Pfeiffer, et al, 2008 and 2010<br>Addgene # 26224
CD8:GFP	CD8:GFP	pJFRC7	UAS	Pfeiffer, et al, 2008 and 2010<br>Addgene # 26220
MET-GAL4	MET-GAL4		-	

Supplementary Table S2. *Drosophila melanogaster* strains used in this study.

Drosophila melanogaster strains	Reference	Source
Cg-GAL4	Asha, et al., 2003	Bloomington Drosophila Stock Center 7011
fru-GAL4	Stockinger, et al., 2005	Barry J. Dickson
GH86-GAL4	Bloomington Drosophila Stock Center	Bloomington Drosophila Stock Center 36339
vGlut-GAL4	Bloomington Drosophila Stock Center	Bloomington Drosophila Stock Center
GMR57C10-GAL4	Jennet, et al., 2012	FlyLight, Janelia Research Campus
SS00324	Jennet, et al., 2012	FlyLight, Janelia Research Campus
LC10a-SS1	Ribeiro, et al., 2018	VDRC
VT043656-GAL4	Tirian and Dickson, 2017	VDRC
VT047880-GAL4	Tirian and Dickson, 2017	VDRC
LC10s-SS2	Ribeiro, et al., 2018	VDRC
UAS-LOV-LexA in attP1 su(Hw)	this study	VDRC, stock number 311200
UAS-eLOV-nls-tdTomato-mLexA:GAD in attP1 su(Hw)	this study	-
UAS-eLOV-nls-tdTomato-mLexA:VP16 in attP1 su(Hw)	this study	-
LexAop-CsChrimson:Venus in attP18	Klapoetke, et al., 2014	Vivek Jayamaran, Janelia Research Campus
LexAop-myr:GFP	Pfeifer, et al., 2010	Gerald Rubin, Janelia Research Campus
y^{1},w^{*};*Mi[MIC]aKap4*^{MI06313} *PVRAP*^{MI06313}	Venken, et al., 2011	Bloomington Drosophila Stock Center 41517
w; P[UAS- Kap-a1.M]2*	Wharton, K. (2008.08.22) personal communication to FlyBase	Bloomington Drosophila Stock Center 25399
w^{1118};*Pbac[IT.GAL4]Kap-*$^{a14018-G4}$/*TM6B, Tb*^{1}	Gohl et al., 2011	Bloomington Drosophila Stock Center 77639

Drosophila melanogaster strains	Reference	Source
y^{1} *w*; Mi[MIC]wrd*^{MI06239} *cdm*^{MI06239}	Nagarkar-Jaiswal, et al., 2015	Bloomington Drosophila Stock Center 59644
w^{1118}; *Mi[ET1]Cse1*^{MB08748} *mdy*^{MB08748}*/SM6a*	Bellen, et al., 2011	Bloomington Drosophila Stock Center 26129
y^{1} *w*; P[w[+mW.hs]=GawB]109(2)80 , P[w[+mC]=UAS-mCD8::GFP.L]LL5*	Lee and Luo, 1999	Bloomington Drosophila Stock Center 8768

Supplementary Table S3. Protocols for blue light delivery used in this study.

	Source of blue light	wavelength	intensity	pulse	Nr of pulses	figures
S2R+ cells	LED	460-500nm	11.7 mW	1Hz for 30s	4 to 6, 1 hour apart	1, S1
Larvae (fat body)	LED	460-500nm	11.7 mW	1Hz for 30s	3 to 6, 30 min apart	2, S2
Pupae (oenocytes)	1-photon	485nm	1.62 µW	0.33Hz for 90s	1	2
Pupae (neurons)	1-photon	458nm	2.38 µW	0.56Hz for 90s	4, 10 min apart	3, S2, S3
Adults (neurons)	1-photon	485nm	2.49 µW	0.33Hz for 90s	4, 10 to 20 min apart	4
